# DSC, TGA-FTIR and FTIR Assisted by Chemometric Factor Analysis and PXRD in Assessing the Incompatibility of the Antiviral Drug Arbidol Hydrochloride with Pharmaceutical Excipients

**DOI:** 10.3390/molecules29010264

**Published:** 2024-01-04

**Authors:** Barbara Rojek, Agata Bartyzel, Wiesław Sawicki, Alina Plenis

**Affiliations:** 1 Department of Analytical Chemistry, Faculty of Pharmacy, Medical University of Gdansk, Gen. J. Hallera 107, 80-416 Gdansk, Poland; 2 Department of General, Coordination Chemistry and Crystallography, Institute of Chemical Sciences, Faculty of Chemistry, Maria Curie-Skłodowska University in Lublin, Maria Curie-Sklodowska Sq. 2, 20-031 Lublin, Poland; agata.bartyzel@mail.umcs.pl; 3 Department of Physical Chemistry, Faculty of Pharmacy, Medical University of Gdansk, Gen. J. Hallera 107, 80-416 Gdansk, Poland; wieslaw.sawicki@gumed.edu.pl

**Keywords:** arbidol hydrochloride, excipients, compatibility/incompatibility, DSC, TGA-FTIR, FTIR, chemometric calculation, PXRD, intrinsic dissolution rate study

## Abstract

Arbidol hydrochloride is an antiviral product widely used in Russia and China for the treatment of, among other diseases, influenza. In recent years, it has turned out to be highly effective against COVID-19. However, there is little knowledge about its physicochemical properties and its behavior in the presence of various pharmaceutical excipients, which could be useful in the development of new preparations by increasing its solubility and bioavailability. For this reason, binary mixtures composed of arbidol hydrochloride and selected pharmaceutical excipients such as chitosan, polyvinylpyrrolione K-30 and magnesium stearate were prepared and subjected to differential scanning calorimetry (DSC), thermogravimetry combined with Fourier transform infrared spectrometry (TGA-FTIR) and Fourier transform infrared spectrometry (FTIR) analyses. In order to obtain clarity in the interpretation of the outcomes, chemometric calculations with factor analysis (FA) were used. Additionally, a powder X-ray diffraction (PXRD) and an intrinsic dissolution rate study were performed for arbidol hydrochloride itself and in the presence of excipients. As a result of the study, it was revealed that arbidol hydrochloride may undergo polymorphic transformations and be incompatible with chitosan and magnesium stearate. However, mixing arbidol hydrochloride with polyvinylpyrrolidone K-30 guarantees the obtaining of durable and safe pharmaceutical preparations.

## 1. Introduction

**Figure 1:**
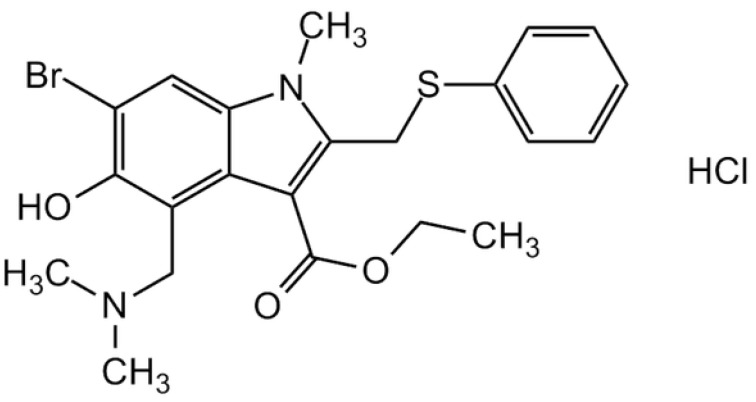
Chemical structure of arbidol hydrochloride.

Arbidol hydrochloride (also called umifenovir hydrochloride) (Figure 1) is a broad-spectrum antiviral agent produced in Russia, used for the prevention and treatment of human lung diseases caused by influenza A and B viruses, hepatitis C virus and other human-pathogenic respiratory viruses [1,2,3]. Since 2020, interest in this drug has increased significantly after its high effectiveness in the fight against the COVID-19 was found. Clinical trials have noted that arbidol hydrochloride can inhibit COVID-19 infection by interfering with the release of SARS-CoV-2 from intracellular vesicles [4].

It turns out that arbidol hydrochloride accelerates the return of fever and the removal of viruses from the respiratory tract and also helps shorten the hospitalization time of COVID-19 patients without major side effects. Moreover, arbidol hydrochloride is one of the least-toxic drugs (LD50 > 4 g/kg), which is associated with no negative impact on the human body at recommended oral doses [3,4,5].

Despite the enormous benefits resulting from the antiviral effect of this drug, there is still very little information in the literature about its physicochemical properties and possible interactions with various excipients. However, it is known that it is a hydrophobic substance, so it would be advisable to select excipients for its formulation that would increase its solubility and, consequently, it bioavailability. Therefore, it would be important to conduct physicochemical tests for this active substance and determine the impact of excipients on its physicochemical properties. Typically, thermal and non-thermal methods are used for physicochemical tests and the compatibility study of the active substance with excipients, most often differential scanning calorimetry (DSC), thermogravimetry (TGA), Fourier transform infrared spectrometry (FTIR) and combined thermal and non-thermal techniques, such as thermogravimetry combined with Fourier transform infrared spectrometry (TGA-FTIR).

DSC and TGA are leading thermal analysis methods used in determining the physicochemical properties of active substances, excipients and in screening the compatibility of an active substance with an excipient [6,7,8]. FTIR spectroscopy, the most frequently used non-thermal method, allows for non-destructive testing of samples at room temperature and is used to confirm the results of thermal analyses [6,7]. In turn, a combination of thermal and non-thermal methods, such TGA-FTIR, is used in the analysis of biologically active compounds to enrich TGA/DSC studies. The results obtained using this method complement the knowledge about the processes occurring under the influence of temperature and provide information about gaseous products released during the heating of compounds, which allows for predicting the mechanism of thermal decomposition of the tested compound.

Taking into account the high antiviral and anti-covid effectiveness and very low toxicity of arbidol hydrochloride, as well as the absence of information on the incompatibility or compatibility of arbidol hydrochloride with various excipients, it seems reasonable to conduct tests on the compatibility of this active substance with excipients and to determine the impact of excipients on its physicochemical properties. DSC, TGA-FTIR and FTIR were used to study the physicochemical properties and compatibility studies of arbidol hydrochloride with various excipients, and chemometric calculations using factor analysis (FA) were applied to clearly estimate the potential incompatibilities or compatibility of this active substance with excipients. Additionally, a powder X-ray diffraction (PXRD) and a test on the release rate of arbidol hydrochloride from mixtures with excipients were performed. Compatibility/incompatibility tests of the hydrochloride with excipients were carried out in the presence of chitosan, magnesium stearate and polyvinylpyrrolidone K-30. The selected excipients are among the most frequently used in solid dosage form technology.

Chitosan is a natural, biocompatible, biodegradable and non-toxic product, and at the same time relatively easy to process with the possibility of creating various chemically and enzymatically modified forms. Due to its ability to bind to cholesterol, fats, proteins and metal ions, it is used orally. Its molecule contains an amino group and numerous hydroxyl groups. Due to the amino group that chitosan has antibacterial properties and has appropriate mucilaginity, which ensures a good connection with the transferred active substance. A large number of functional groups enable easy modification of chitosan and ensure high solubility in an acidic environment. In turn, magnesium stearate is widely used in the production of dietary supplements and medicines in the form of tablets, capsules and powders. It is a good lubricant and prevents drugs from sticking to the surface of the equipment during their production, as well as the drug ingredients from sticking together inside the capsule. The use of magnesium stearate affects the quality and appropriate properties of manufactured drugs; this compound delays their decomposition, so they are absorbed in the right place in the digestive tract. As an excipient, it may enhance therapeutic effects. This improves absorption, solubility and consistency. Polyvinylpyrrolidon K-30 is used in the pharmaceutical industry as a filler or binder, which helps improve the quality of medicinal products. It is also used as a carrier of synthetic polymers for dispersing and suspending drugs [7].

## 2. Results

### 2.1. DSC Study with FA Calculation

DSC as a screening method enables fast identification of potential incompatibilities between the ingredients of pharmaceutical preparations by conducting analysis at elevated temperatures. Generally, the DSC curves of mixtures are compared with those of the ingredients. Incompatibility between ingredients is noticeable on the DSC curve of the mixture by the absence of the characteristic peak(s) of the ingredient(s), as well as a change in the shape of the peak(s) and a significant shift in the extreme of the peak(s) or the appearance of a new peak(s) that does not originate from the ingredient(s).

**Figure 2:**
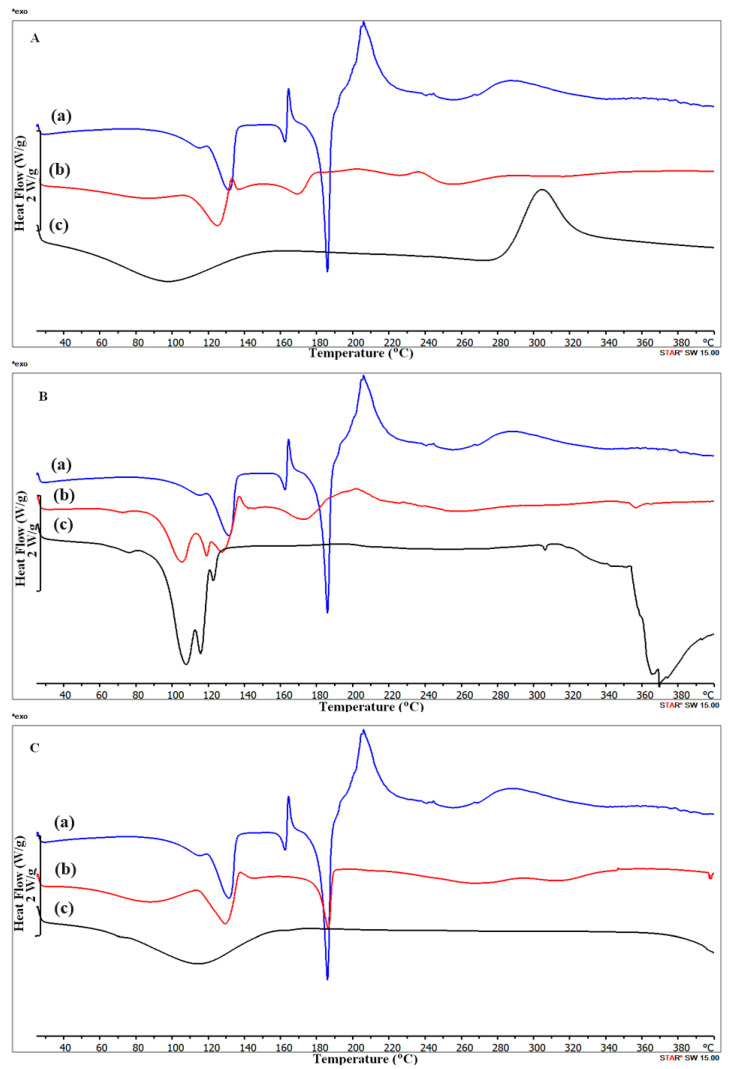
DSC curves of (**A**) (**a**) arbidol hydrochloride, (**c**) chitosan and (**b**) its mixture at 1:1 (*w/w*) ratio; (**B**) (**a**) arbidol hydrochloride, (**c**) magnesium stearate and (**b**) its mixture at 1:1 (*w/w*) ratio; and (**C**) (**a**) arbidol hydrochloride, (**c**) polyvinylpyrrolidone K-30 and (**b**) its mixture at 1:1 (*w/w*) ratio.

The results of DSC analyses of arbidol hydrochloride, excipients and their mixtures are presented in Figure 2 and collected in Table 1.

The DSC curve of arbidol hydrochloride (Figure 2Aa,Ba,Ca) showed the complexity of phase transformations occurring in this substance under the influence of elevated temperature. In the temperature range of 90–150 °C, an endothermic effect was observed with two overlapping peaks with extremes at 114.46 °C and 131.48 °C, with a total heat of transformation of −125.93 J/g, indicating the release of crystallization water [9]. Moreover, the endothermic peak at 162.27 °C turning into an exothermic peak at 164.29 °C may indicate a polymorphic transition, during which one of the forms melts, followed by “cold crystallization”, after which the other polymorphic form melts at 185.77 °C [9] and decomposes at 205.79 °C.

Chitosan subjected to DSC analysis (Figure 2Ac) is initially dehydrated in the temperature range of approx. 25–140 °C. After dehydration, it undergoes a glass transformation at a temperature of approx. 273 °C and decomposes at 305 °C [10]. In turn, polvinylpyrrolidone K-30 (Figure 2Cc), after dehydration at 91.3 °C, begins to soften, and then decomposes at temperatures above 300 °C.

A different course of the DSC curve is observed in the case of magnesium stearate (Figure 2Bc), which is not a polymer compound but a salt composed of a mixture of fatty acids, in which the largest share is stearate and palmitate.

Magnesium stearate has several crystalline forms and potentially an amorphous form [11]. At a temperature of 75.75 °C, magnesium stearate dehydrates and decomposes [12], transforming into an anhydrous form, which melts at 107.37 °C [12,13], and the so-called reverse micellar phase [12]. Further, at 115.73 °C, the micellar phase transforms into a hexagonal mesostructure [12], and at 122.64 °C, another transformation of the anhydrous form occurs [11]. In turn, at a temperature of approx. 173 °C, a very low-energy endothermic transformation was observed, which was attributed to the disintegration of the hexagonal form and the formation of another so-called reverse micellar phase [12]. It is also believed that one form of magnesium stearate is stable up to 250 °C [12]. However, above 300 °C, magnesium stearate decomposes.

In the case of all mixtures of arbidol hydrochloride with excipients (Figure 2Ab,Bb,Cb), it is possible to notice the lack of endothermic and exothermic peaks in the range of 160–170 °C, which are attributed to the polymorphic transformation of arbidol hydrochloride, or these peaks overlapped with the melting peak of the second polymorphic form, creating a broad endothermic peak with a clear extreme at approx. 185 °C [13], or these peaks were shifted to the range of 130–140 °C. The DSC curve of the binary mixture of arbidol hydrochloride with polyvinylpyrrolidone K-30 shows a broad, diffuse endothermic peak in the range of 25–100 °C, reflecting the dehydration of the excipient and a broad endothermic peak at approx. 100–135 °C related to the release of crystallization water from arbidol hydrochloride. Moreover, in the range of 130–140 °C, a polymorphic transformation of arbidol hydrochloride probably occurs. However, in the range of 140–190 °C, a broad endothermic peak can be observed with a clearly narrow extreme at approx. 185 °C, attributed to the melting of the polymorphic form of arbidol hydrochloride, which then turns into an exothermic peak indicating the decomposition of the substance. It should be noted that the characteristic peak of arbidol hydrochloride at 185 °C in this mixture was retained. It can therefore be assumed that arbidol hydrochloride in a mixture with polyvinylpyrrolidone K-30 is probably compatible. Incompatibilities may occur in mixtures of arbidol hydrochloride with chitosan (Figure 2Ab) and magnesium stearate (Figure 2Bb) because in these mixtures, the crystallinity of arbidol hydrochloride decreases and the extremum of the melting peak of this substance shifts from 185 °C to approximately 175 °C and widens.

**Figure 3:**
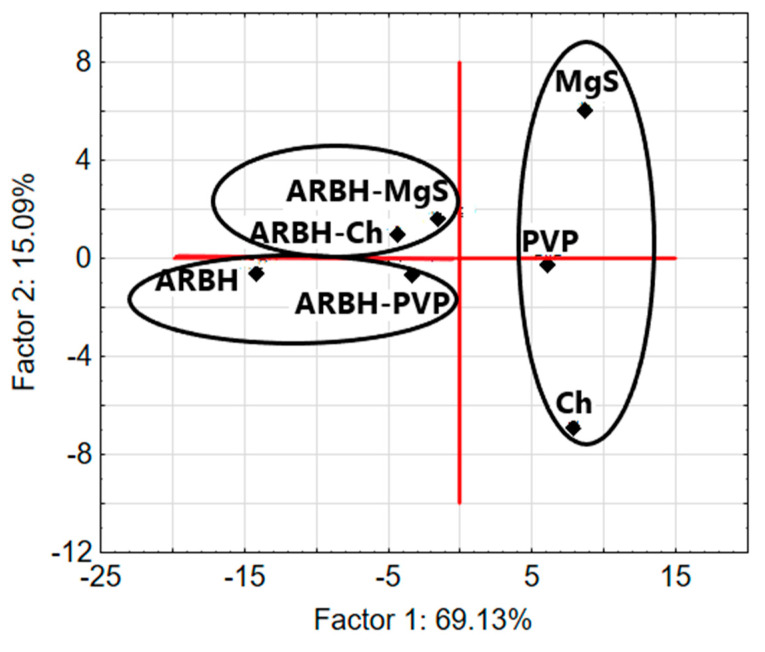
FA score biplot for the first two factors based on DSC data for arbidol hydrochloride (ARBH), chitosan (Ch), magnesium stearate (MgS), polyvinylpyrrolidone K-30 (PVP) and their binary mixtures.

Transparency in the interpretation of DSC data was achieved by using chemometric factor analysis (FA) (Figure 3). Factor analysis is an unsupervised learning technique that aims to reduce the dimensionality of large datasets. It increases interpretability while minimizing information loss [14]. As shown in Figure 3, there are three clusters located in the range of Factor 1 values, between −15 and 10. One of the clusters contains arbidol hydrochloride and a mixture of arbidol hydrochloride with polyvinylpyrrolidone K-30. This indicates the similarity of the DSC curves of arbidol hydrochloride and its mixture with polyvinylpyrrolidone K-30, and at the same time, it confirms the compatibility between the ingredients of this mixture. Three excipients can be found in another cluster, demonstrating the similarity of their DSC curves. In the next cluster, you can see two mixtures of arbidol hydrochloride with chitosan and magnesium starate.

### 2.2. TGA-FTIR Experiments

**Figure 4:**
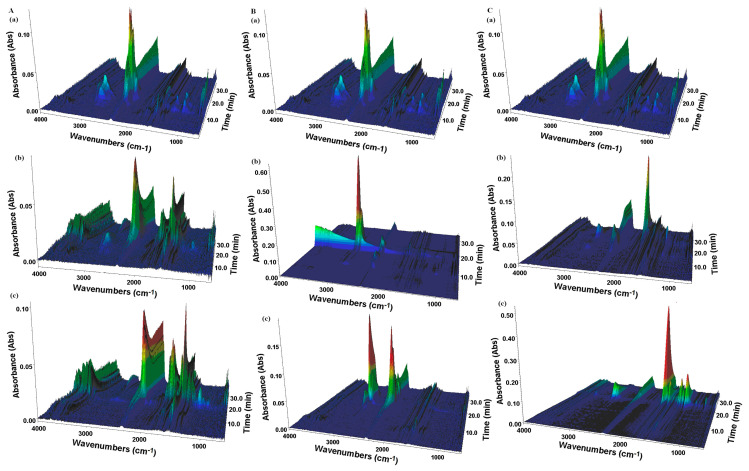
3D surface plot for TGA-FTIR spectra of the evolved gaseous products for (**A**) (**a**) arbidol hydrochloride, (**c**) chitosan and (**b**) its mixture at 1:1 (*w/w*) ratio; (**B**) (**a**) arbidol hydrochloride, (**c**) magnesium stearate and (**b**) its mixture at 1:1 (*w/w*) ratio; and (**C**) (**a**) arbidol hydrochloride, (**c**) polyvinylpyrrolidone K-30 and (**b**) its mixture at 1:1 (*w/w*) ratio.

**Figure 5:**
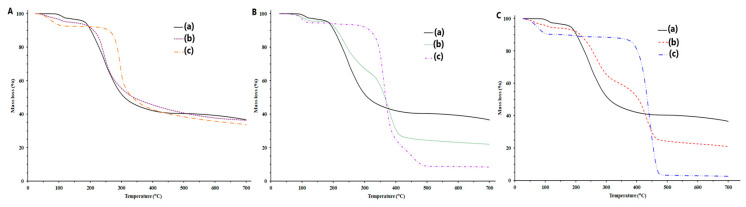
TGA curves of (**A**) (**a**) arbidol hydrochloride, (**c**) chitosan and (**b**) its mixture at 1:1 (*w/w*) ratio; (**B**) (**a**) arbidol hydrochloride, (**c**) magnesium stearate and (**b**) its mixture at 1:1 (*w/w*) ratio; and (**C**) (**a**) arbidol hydrochloride, (**c**) polyvinylpyrrolidone K-30 and (**b**) its mixture at 1:1 (*w/w*) ratio.

TGA-FTIR analysis of arbidol hydrochloride revealed that the substance is stable up to approximately 90 °C in an inert atmosphere, above which it gradually decomposes (Figure 4 and Figure 5). In the first stage, the ester bond is probably broken, which indicates the presence in the FTIR spectrum of gas products from the bands characteristic of water (3450–4000 cm^−1^ and 1300–1950 cm^−1^) and carbon dioxide (2240–2400 cm^−1^) (Figure 4). Upon further heating, the compound undergoes defragmentation and pyrolysis, which results in the release of water and carbon dioxide molecules, and at a temperature of approximately 155 °C, weak bands characteristic of methanol occur (3150–2750 cm^−1^ and 1100–950 cm^−1^).

Above 180 °C, a series of bands appear at 3069, 2965, 1270, 1179, 1066, 1026, 739, 692, 669 and 650 cm^−1^ (Figure 4) probably reflecting the release of mainly thiophenol molecules, aliphatic amines and benzene during decomposition. The intensity of the above bands decreases at a temperature of about 310 °C, as the characteristic vibrations of ammonia molecules become visible (doublet 965 and 929 cm^−1^) and above 380 °C for methane (a series of bands in the range of 3150–2700 cm^−1^ with a characteristic maximum at 3015 cm^−1^). In turn, at a temperature of 530 °C, CO and HCl vibration bands (2650–3100 cm^−1^) are observed in the spectrum.

In the case of the thermal decomposition of auxiliary substances, it can be noticed that in the initial phase, there is a mass loss of several percent related to the dehydration process. In the next stage of decomposition, thermal destruction takes place, resulting in the formation of intermediate products.

Finally, the carbonization residues are combusted. In the case of TGA curves of mixtures of arbidol hydrochloride with excipients (Figure 5), sections similar to the TGA curve of arbidol hydrochloride alone can be seen. However, in the case of three-dimensional surface plots of TGA-FTIR spectra (Figure 4) for the mixture of arbidol hydrochloride with polyvinylpyrrolidone K-30, convergence with those of arbidol hydrochloride itself was observed.

### 2.3. FTIR Results with FA Calculation

**Figure 6:**
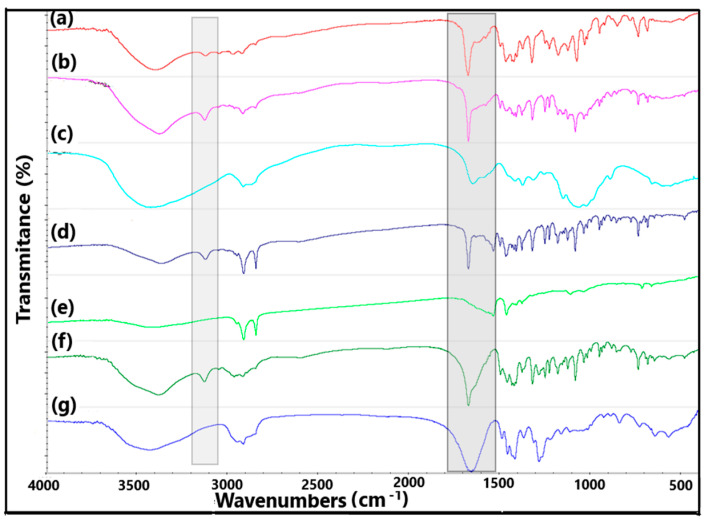
FTIR spectra of (a) arbidol hydrochloride, (b) mixture with chitosan, (c) chitosan, (d) mixture with magnesium stearate, (e) magnesium stearate, (f) mixture with polyvinylpyrrolidone K-30 and (g) polyvinylpyrrolidone K-30.

The spectra of active substance and excipients are shown in Figure 6. In the case of arbidol hydrochloride (Figure 6a), a broad, intense band at 3407 cm^−1^ corresponding to OH stretching vibrations can be seen. However, the absorption band at 3126 cm^−1^ is assigned to aromatic CH stretching vibrations [13] and also to NH stretching vibrations [15]. The next absorption band at 3056 cm^−1^ corresponds to the aromatic vibrations of CH [16]. In turn, the absorption bands at 2980 cm^−1^, 2926 cm^−1^, 2853 cm^−1^ and 2708 cm^−1^ reflect the vibrations of the CH_3_ and CH_2_ groups [16], and the band at 1682 cm^−1^ is assigned to the C=O stretching vibrations [13].

Chitosan (Figure 6c), which belongs to carbohydrate polymers, exhibits absorption of radiation from OH and CH stretching vibrations in the wave number range of 3600–2800 cm^−1^, and in the range of 1500–1200 cm^−1^ for the HCH and CH_2_OH groups and in the range of 1200–950 cm^−1^ for the CO stretching groups. However, in the spectroscopic region of 950–700 cm^−1^, there are bands of deformational vibrations of the COH, CCH and OCH groups, and in 700–500 cm^−1^, there are bands of exocyclic deformation vibrations (CCO), and below 500 cm^−1^, endocyclic deformation vibrations (CCO, CCC) [17,18]. The spectrum of polyvinylpyrrolidone K-30 (Figure 6g) shows a broad band of OH stretching vibrations at 3400 cm^−1^, then an overlapping band at approximately 2900 cm^−1^ assigned to CH stretching vibrations and an intense band at 1645 cm^−1^ responsible for amide I vibrations inline. Moreover, in the spectral range 1490–1420 cm^−1^, CH deformation vibrations occur. However, the bands at 1280 cm^−1^ and around 1020 cm^−1^ were assigned to NC stretching vibrations [19]. In turn, in the case of magnesium stearate (Figure 6e), there is a wide band at 3452 cm^−1^ of OH stretching vibrations associated with the water molecule. The bands at 2917 cm^−1^ and 2850 cm^−1^ are assigned to CH stretching vibrations. However, two “twin” bands at 1577 cm^−1^ and 1466 cm^−1^ reflect the asymmetric stretching and symmetric stretching vibrations of the COO^−^ groups [20].

In the case of a mixture of arbidol hydrochloride and magnesium stearate (Figure 6d), it can be seen that the NH band coming from the active substance at 3126 cm^−1^ increased in intensity, which may indicate the formation of hydrogen bonds of the NH group with the COO^−^ magnesium stearate group. Moreover, the CO vibration band of arbidol hydrochloride at 1682 cm^−1^ increased in intensity and its width decreased. Changes in the absorption bands of the active substance can also be seen in the wavenumber range 1650–600 cm^−1^. Changes in the absorption bands of arbidol hydrochloride are also noticeable in the same spectral ranges as above for mixtures of arbidol hydrochloride with chitosan (Figure 6b) and polyvinylpyrrolidone (Figure 6f).

**Figure 7:**
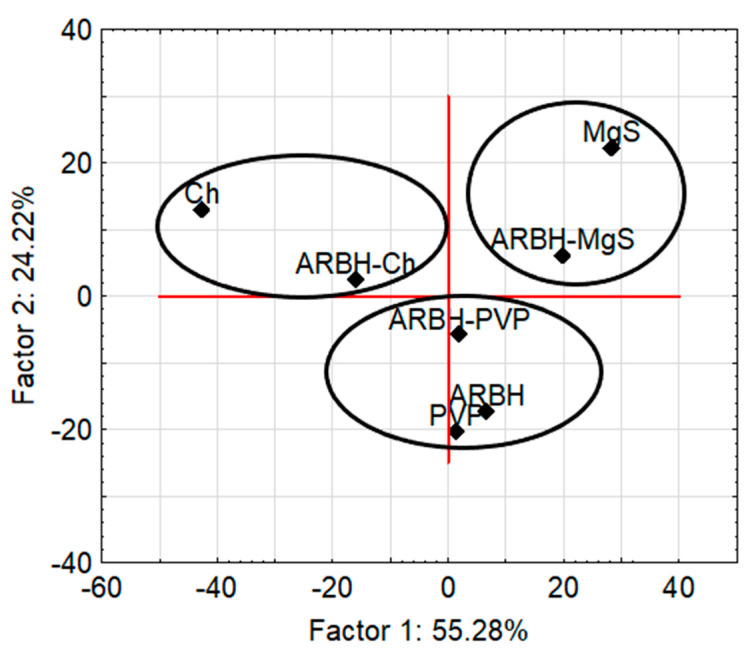
FA score biplot for the first two factors based on FTIR data for arbidol hydrochloride (ARBH), chitosan (Ch), magnesium stearate (MgS), polyvinylpyrrolidone K-30 (PVP) and their binary mixtures.

To improve the interpretation of FTIR data, chemometric factor analysis (FA) was used. Three clusters can be seen in the two-dimensional plot of Factor 1 and Factor 2 presented in Figure 7.

One of the clusters is formed by arbidol hydrochloride, polyvinylpyrrolidone K-30 and a mixture thereof. This proves the similarity between the FTIR spectrum of the arbidol hydrochloride mixture and the spectra of the ingredients. In turn, the other two clusters contain a mixture of arbidol hydrochloride and an excipient that is an ingredient of this mixture. Summarizing the DSC and FTIR results supported by the chemometric method—factor analysis (Table 2)—it can be concluded that only polyvinylpyrrolidone K-30 is compatible with arbidol hydrochloride; therefore, this excipient can be mixed in the pharmaceutical formulation together with arbidol hydrochloride.

### 2.4. PXRD Study

**Figure 8:**
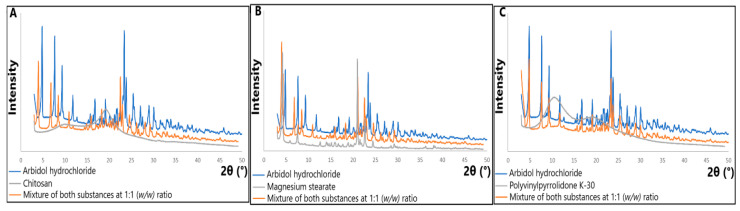
PXRD patterns for (**A**) arbidol hydrochloride, chitosan and its mixture at 1:1 (*w*/*w*) ratio; (**B**) arbidol hydrochloride, magnesium stearate and its mixture at 1:1 (*w*/*w*) ratio; and (**C**) arbidol hydrochloride, polyvinylpyrrolidone K-30 and its mixture at 1:1 (*w*/*w*) ratio.

PXRD studies performed at room temperature (Figure 8) disclosed the crystalline forms of arbidol hydrochloride and magnesium stearate. The most pronounced diffraction lines in the PXRD pattern of arbidol hydrochloride occur at 2θ of 4.80; 7.66; 9.35; 23.42; 23.83, and also at 2θ of 11.79; 16.81; 19.13; 25.39; 27.08; 29.03 and 30.05. In turn, in the PXRD pattern of magnesium stearate (Figure 8B), diffraction lines of higher intensity are observed at 4.99; 8.29; 21.81 and 23.45. However, the PXRD patterns of chitosan and polyvinylpyrrolidone K-30 (Figure 8A,C) reveal their amorphous nature.

PXRD analyzes of arbidol hydrochloride mixtures showed that the patterns of mixtures with chitosan and magnesium stearate consist of diffraction peaks related to the diffraction peaks of arbidol hydrochloride and excipient, but the diffraction peaks of the arbidol hydrochloride in the patterns of these mixtures compared to the pattern of arbidol hydrochloride alone are shifted towards lower 2θ values. Only in the PXRD pattern of the mixture with polyvinylpyrrolidone K-30 (Figure 8C), the diffraction peaks of arbidol hydrochloride in the mixture coincide with the diffraction peaks of arbidol hydrochloride itself. This suggests no changes in the structure of the active substance after mixing with polyvinylpyrrolidone K-30.

### 2.5. Intrinsic Dissolution Study

**Figure 9:**
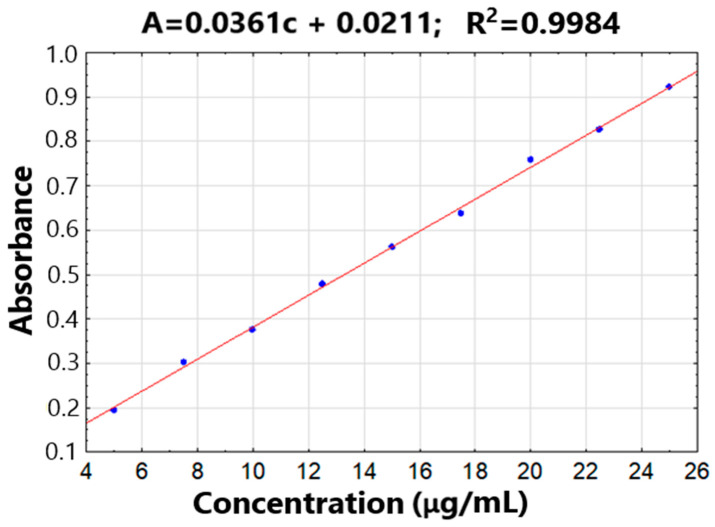
The calibration curve for dissolution study of arbidol hydrochloride.

The results of the intrinsic rate of dissolution study for arbidol hydrochloride and its formulations with excipients were summarized in Table 3. The calibration curve for dissolution study of arbidol hydrochloride is presented in Figure 9. Due to the disintegration of the arbidol hydrochloride disc, it was not possible to determine the release rate of arbidol hydrochloride from its mixtures. Moreover, the disc of the mixture of arbidol hydrochloride with polyvinylpyrrolidone K-30 also disintegrated. Mixtures of arbidol hydrochloride with chitosan and magnesium stearate did not decompose during the test.

According to the literature data, chitosan is used primarily as a coating agent, im-forming agent and mucoadhesive, while magnesium stearate is used as a tablet and capsule lubricant and polyvinylpyrrolidone are used as a disintegrant, dissolution enhancer or suspending agent, and tablet binder [21].

## 3. Materials and Methods

### 3.1. Chemicals

Arbidol hydrochloride (purity ≥ 98%) from Sigma-Aldrich (Steinhem, Germany), chitosan (low-viscous, purity ≥ 95%) and polyvinylpyrrolidone K-30 (purum) from Fluka (Siegen, Germany) and magnesium stearate (purity Ph. Eur.) from Faci (Carasco Genoa, Italy) were used. 

### 3.2. Preparation of Arbidol Hydrochloride Mixtures

Three binary mixtures of arbidol hydrochloride with excipients (chitosan, polyvinylpyrrolidone K-30 and magnesium stearate) at a 1:1 (*w*/*w*) ratio were obtained in a porcelain mortar by homogenizing the constituents using a plastic spatula.

Preparing mixtures at a 1:1 (*w*/*w*) ratio ensures an increase in the probability of interactions, as thermal events are greater when the excipient content is higher; hence, the probability of detecting potential interactions increases [22].

### 3.3. Differential Scanning Calorimetry (DSC)

The DSC experiments were performed using the heat-flux DSC 822^e^ Mettler Toledo apparatus (Schwerzenbach, Switzerland) in nitrogen (99.998% of purity) at a 70 mL/min flux rate and in the temperature range of 25–400 °C at 10 °C heating rate per min with a 4.25 ± 0.20 mg sample placed in a 40 μL aluminum pan with two holes in the lid, with the empty pan being the reference sample. The measurements were taken in triplicate and controlled by STARe 15 software. The instrument was calibrated for indium (99.999% purity) and zinc (99.998% purity) obtained from Mettler Toledo (Schwerzenbach, Switzerland).

### 3.4. Thermogravimetry Coupled with FTIR Spectroscopy (TG-FTIR)

Thermogravimetric analysis of compounds combined with the identification of gaseous decomposition products (TG-FTIR) was carried out using a TG Q5000 thermobalance (TA Instruments, New Castle, DE, USA) coupled with a Nicolet 6700 FTIR spectrophotometer (Thermo Scientific, Madison, WI, USA). Samples of 19.70–21.43 mg placed in open platinum crucibles were heated in the temperature range of 25–700 °C (20 °C heating rate per min) in nitrogen with a flow rate of 50 mL/min. In parallel with the thermal analysis, FTIR spectra of gaseous products in the range of 4000–600 cm^−1^ with a resolution of 4 cm^−1^ were recorded (n = 3).

### 3.5. FTIR Spectroscopy (FTIR)

The FTIR measurements were taken using a Nicolet 380 FTIR spectrometer with OMINIC software version 8.2.387 from Thermo Fisher Scientific (Madison, WI, USA). A 1 mg sample was mixed with 100 mg KBr, compressed into a tablet, and the spectrum was recorded in the range of 4000–400 cm^−1^ with a resolution of 4 cm^−1^, averaging over 16 scans and also recording the background spectrum (n = 3).

### 3.6. Chemometric Factor Analysis (FA)

All measurements were performed in triplicate and, after averaging, were subjected to FA calculations in Statistica 13.3 (StatSoft Inc., Tulsa, OK, USA). Matrices for calculations were constructed using thermal (obtained from DSC curves) and spectral (obtained from FTIR spectra) data for arbidol hydrochloride, excipients and its binary mixtures, which were standardized using the Standard Normal Variate (SNV) algorithm [23,24].

The matrix composed of DSC data included the heat values in the range of 25–400 °C. However, the matrix with FTIR data contained the transmittance values from the wave number ranges of 3800–2600 cm^−1^ and 1900–1000 cm^−1^ at a resolution of 4 cm^−1^.

Table 4 shows the values of the first three factors (Factor 1, Factor 2 and Factor 3), calculated for DSC and FTIR data sets. Taking into account the fact that the first two factors explain more than 84% of the total variance for the DSC data and more than 79% of the total variance for the FTIR data, respectively, sample classification was performed using a two-dimensional score plot (Factor 1 and Factor 2).

### 3.7. Powder X-ray Diffraction (PXRD)

PXRD analyzes for arbidol hydrochloride, excipients and their mixtures were performed using a Philips X’Pert PRO X-ray diffraction system (Almelo, The Netherlands) in the 2theta range of 5–50° with Cu-Kα (λ = 1.541 Å); setting the X-ray tube at 30 mA and 40 kV, the counting time was 3 s per step and the counting step (2θ) was 0.02°. The calibration was carried out with a polycrystalline silicon standard.

### 3.8. Intrinsic Dissolution Test

The intrinsic dissolution tests were performed based on USP-35 <1087> chapter (“Apparent intrinsic dissolution-dissolution testing procedures for rotating disk and stationary disk”) [25] and the protocol provided by Ibraheem and Wagner [26], in which 100 mg of arbidol hydrochloride or its binary mixtures with excipients at a 1:1 (*w/w*) ratio were placed into the compact matrix assembly (0.4 cm in diameter) of the rotating disk and compressed at a pressure of 10 kN for 2 min. The tests were carried out at a temperature of 37 ± 0.5 °C in 500 mL of 0.1 M hydrochloric acid as the dissolving medium, with a stirring rate of 50 rpm., within 60 min using a USP DIS 6000i dissolution apparatus (Copley Scientific, Nottingham, UK). Samples of 5 mL were taken in triplicate at appropriate time points, filtered through a 0.45 µm Chromafil PET-45/25 filter (Alchem, Torun, Poland), and spectrophotometric measurements were made in triplicate at the maximum absorbance wavelength of 258 nm [13] using a UV-1600 spectrophotometer (AOE Instruments, Shanghai, China).

## 4. Conclusions

The results obtained using thermal and non-thermal instrumental techniques for arbidol hydrochloride and its mixtures with excipients provided significant information on the physicochemical properties of the active substance and indicated excipients that may influence the change of its physicochemical properties and, therefore, its bioavailability. PXRD analysis of arbidol hydrochloride mixtures showed that only in the mixture with polyvinylpyrrolidone K-30 were there no changes in the structure of the active substance.

Analyses performed using thermal techniques allowed for the determination of the temperatures at which the active substance is thermally stable and enabled the identification of phase changes occurring in this substance. However, with the use of non-thermal techniques such as FTIR spectroscopy, it was possible to verify the incompatibilities detected using DSC and PXRD to determine whether these incompatibilities also occur immediately after mixing the ingredients at room temperature and what chemical groups may be the cause of the incompatibilities.

As a result of the research, it was revealed that arbidol hydrochloride may undergo polymorphic transformations and be incompatible in mixtures with two excipients, i.e., chitosan and magnesium stearate. However, mixing arbidol hydrochloride with polyvinylpyrrolidone K-30 guarantees the obtaining of durable and safe pharmaceutical preparations.

## Figures and Tables

**Table 1:** DSC results of arbidol hydrochloride and excipients.

Substance	T Onest (°C)	T Peak(s) (°C)	Enthalpy (J/g)
Arbidol hydrochloride	113.89	114.46; 131.48	−125.93
	156.67	162.27; 164.29	−25.47; 16.28
	180.37	185.77	−186.18
	198.08	205.79	108.79
	273.88	287.97	45.86
Chitosan	92.72	93.78	−280.91
	194.93	273.02	−112.89
	286.44	305.21	250.18
Magnesium stearate	64.84	75.75; 107.37; 115.73;	−321.22
	304.23	122.64	−42.51
	358.78	255.17; 306.24; 368.6	
Polyvinylpyrrolidone K-30	48.0	91.3	−292.1

**Table 2:** Compatibility/incompatibility prediction for arbidol hydrochloride mixtures based on DSC and FTIR with the FA approach.

Arbidol Hydrochloride Mixed with	DSC and FTIR with Aid of FA Approach
Chitosan	Incompatibility
Magnesium stearate	Incompatibility
Polyvinylpyrrolidone K-30	Compatibility

**Table 3:** Results of intrinsic dissolution study.

Formulation	Stability of the Tablet in 0.1 M HCl	Amount of Arbidol Hydrochloride Released after 60 min
Arbidol hydrohloride	disintegration after 5 min	-
Arbidol hydrochloride with chitosan	>60 min	~40%
Arbidol hydrochloride with magnesium stearate	>60 min	~20%
Arbidol hydrochloride with polyvinylpyrrolidone K-30	disintegration after 1 min	-

**Table 4:** Values of the first three factors (Factor 1, Factor 2 and Factor 3) calculated for DSC and FTIR data sets.

Factors Variance (%)	Methods	
	DSC	FTIR
Factor 1	69.13	55.28
Factor 2	15.09	24.22
Factor 3	8.55	10.34

## Data Availability

Data are contained within the article.

## References

[1] Zhu Z., Lu Z., Xu T., Chen C., Yang G., Zha T., Lu J., Xue Y. (2020). Arbidol monotherapy is superior to lopinavir/ritonavir in treating COVID-19. J. Infect..

[2] Deng L., Li C., Zeng Q., Liu X., Li X., Zhang H., Hong Z., Xia J. (2020). Arbidol combined with LPV/r versus LPV/r alone against Corona Virus Disease 2019: A retrospective cohort study. J. Infect..

[3] Manin A.N., Surov A.O., Churakov A.V., Perlovich G.L. (2015). Crystal structures, thermal analysis, and dissolution behavior of new solid forms of the antiviral drug arbidol with dicarboxylic acids. Crystals.

[4] Wang X., Cao R., Zhang H., Liu J., Xu M., Hu H., Li Y., Zhao L., Li W., Sun X. (2020). The anti-influenza virus drug, arbidol is an efficient inhibitor of SARS-CoV-2 in vitro. Cell Discov..

[5] Glushkov R.G. (1992). Arbidol antiviral, immunostimulant, interferon inducer. Drug Future.

[6] Chadha R., Bhandari S. (2014). Drug-excipient compatibility screening. Role of thermoanalytical and spectroscopic techniques. J. Pharm. Biomed. Anal..

[7] Adeyeye M.C., Brittain H.G. (2008). Preformulation in Solid Dosage form Development.

[8] Craig D.Q.M., Reading M. (2007). Thermal Analysis of Pharmaceuticals.

[9] Wang L., Sun Y., Kuang C., Zhang X. (2015). Preparation and evaluation of taste masked oral suspension of arbidol hydrochloride. Asian. J. Pharm. Sci..

[10] Rojek B., Wesolowski M. (2017). Compatibility studies of hydrocortisone with excipients using thermogravimetric analysis supported by multivariate statistical analysis. J. Therm. Anal. Calorim..

[11] Delaney S.P., Nethercott M.J., Mays C.J., Winquist N.T., Arthur D., Calahan J.L., Sethi M., Pardue D.S., Kim J., Amidon G. (2017). Characterization of synthesized and commercial forms of magnesium stearate using differential scanning calorimetry, thermogravimetric analysis, powder x-ray diffraction, and solid-state NMR spectroscopy. J. Pharm. Sci..

[12] Haware R.V., Vinjamuri B.P., Sarkar A., Stefik M., Stagner W.C. (2018). Deciphering magnesium stearate thermotropic behavior. Inter. J. Pharm..

[13] Anwer M.K., Iqbal M., Ahmed M.M., Aldawsari M.F., Ansari M.N., Ezzeldin E., Khalil N.Y., Ali R. (2021). Improving the solubilization and bioavailability of arbidol hydrochloride by the preparation of binary and ternary β-cyclodextrin complexes with Poloxamer 188. Pharmaceuticals.

[14] Miller J.N., Miller J.C. (2010). Statistics and Chemometrics for Analytical Chemistry.

[15] Li X., Wang X., Jiang Q., Chi F., Liu Q., Zhang T. (2017). The delivery of arbidol by salt engineering: Synthesis, physicochemical properties and pharmacokinetics. Drug Dev. Ind. Pharm..

[16] Silverstein R.M., Webster F.X., Kiemle D.J. (2005). Spectrometric Identification of Organic Compounds.

[17] Černá M., Barros A.S., Nunes A., Rocha S.M., Delgadillo I., Čopíková J., Coimbra M.A. (2003). Use of FT-IR spectroscopy as a tool for the analysis of polysaccharide food additives. Carbohydr. Polym..

[18] Kačuráková M., Wilson R.H. (2001). Developments in mid-infrared FT-IR spectroscopy of selected carbohydrates. Carbohydr. Polym..

[19] Mireles L.K., Wu M.R., Saadeh N., Yahia L., Sacher E. (2020). Physicochemical characterization of polyvinyl pyrrolidone: A tale of two polyvinyl pyrrolidones. ACS Omega.

[20] Nep E.I., Conway B.R. (2012). Preformulation studies on grewia gum as a formulation excipient. J. Therm. Anal. Calorim..

[21] Rowe R.C., Sheskey P.J., Quinn M.E. (2009). Handbook of Pharmaceutical Excipients.

[22] Ali F., Kumar R., Lal Sahu P., Nath Singh G. (2017). Physicochemical characterization and compatibility study of roflumilast with various pharmaceutical excipients. J. Therm. Anal. Calorim..

[23] Rojek B., Wesolowski M. (2019). FTIR and TG analyses coupled with factor analysis in a compatibility study of acetazolamide with excipients. Spectrochim. Acta.

[24] Rinan Å., Berg F., Engelsen S.B. (2009). Review of the most common pre-processing techniques for near-infrared spectra. TrAc Trends Anal. Chem..

[25] (2015). The United States Pharmacopeia, USP 39, NF 34. Rockville.

[26] Ibraheem B., Wagner K.G. (2021). Influence of high pressure compaction on solubility and intrinsic dissolution of ibuprofen binary mixtures employing standard excipients. Int. J. Pharm. X.

